# Does Maternal Vitamin D Deficiency Increase the Risk of Preterm Birth: A Meta-Analysis of Observational Studies

**DOI:** 10.3390/nu8050301

**Published:** 2016-05-20

**Authors:** Lu-Lu Qin, Fang-Guo Lu, Sheng-Hui Yang, Hui-Lan Xu, Bang-An Luo

**Affiliations:** 1 Department of Prevention Medicine and Pathogenic Biology, Medical School, Hunan University of Chinese Medicine, Changsha 410208, China; powerestlulu@163.com (L.-L.Q.); lufangguo0731@163 (F.-G.L.); 2289836475@126.com (S.-H.Y.); 2 Department of Social Medicine and Health Management, Xiangya School of Public Health, Central South University, Changsha 410078, China; huilanxu1963@sina.com; 3 Department of Mental Health, Brain Hospital of Hunan Province, Changsha 410007, China

**Keywords:** vitamin D deficiency, preterm birth, pregnant women, meta

## Abstract

There are disagreements among researchers about the association between vitamin D deficiency during pregnancy and preterm birth (PTB). Therefore, we conducted a meta-analysis of observational studies to evaluate this association. We performed a systematic literature search of PubMed, MEDLINE and the Cochrane Library through August 2015 with the following keywords: “vitamin D” or “cholecalciferol” or “25-hydroxyvitamin D” or “25(OH)D” in combination with “premature birth” or “preterm birth” or “PTB” or “preterm delivery” or “PTD” or “prematurity”. Our meta-analysis of 10 studies included 10,098 participants and found that pregnant women with vitamin D deficiency (maternal serum 25 (OH) D levels < 20 ng/mL) experienced a significantly increased risk of PTB (odds ratio (OR) = 1.29, 95% confidence intervals(CI): 1.16, 1.45) with low heterogeneity (*I*^2^ = 25%, *p* = 0.21). Sensitivity analysis showed that exclusion of any single study did not materially alter the overall combined effect. In the subgroup analyses, we found that heterogeneity was obvious in prospective cohort studies (*I*^2^ = 60%, *p* = 0.06). In conclusion, pregnant women with vitamin D deficiency during pregnancy have an increasing risk of PTB.

## 1. Introduction

Preterm birth (PTB) is the birth of a baby before 37 complete weeks of gestation. Every year, 15 million neonates worldwide are born preterm [1]. PTB, as the largest cause of neonatal deaths worldwide [2], puts surviving children at risk for cerebral palsy, behavioral problems, bronchopulmonary dysplasia, retinopathy of prematurity, hearing impairments, increased hospital admissions and academic underachievement [3].

Vitamin D is a fat-soluble metabolite required for the proper regulation of many body systems, as well as for normal human growth and development. Considered as a common and thorny public health problem around the world [4,5,6], especially for pregnant women, maternal vitamin D deficiency, or insufficiency, has been demonstrated to be related to a variety of adverse maternal and fetal outcomes, including gestational diabetes mellitus (GDM), preeclampsia, small for gestational age (SAG) and other tissue-specific conditions [6].

There have been some studies describing the association between maternal vitamin D levels and preterm birth; however, their results are conflicting. According to some studies, vitamin D deficiency during pregnancy is associated with an increasing risk of preterm birth. Wagner *et al.* [7] reported that pregnant women with serum concentrations of vitamin D less than 20 ng/mL had 3.81 times the odds of a preterm birth compared to those with serum concentrations of vitamin D greater than 40 ng/mL. Bodnar *et al.* [8] reported that the risk of preterm birth decreased significantly as serum concentrations of 25-hydroxyvitamin D increased to approximately 36 ng/mL and then plateaued. As reported by Shibata *et al.* [9], lower levels of vitamin D among pregnant women are associated with premature delivery in Japan. In contrast, Zhou *et al.* [10] reported that vitamin D deficiency was associated with a reduced risk of preterm birth. They found that the prevalence of preterm delivery in the high-level (maternal 25(OH)D ≥ 30 ng/mL) was higher than that in the low (≤20 ng/mL) and medium (20–30 ng/mL) level groups in southern China. However, other studies have demonstrated no association between vitamin D status and preterm birth. Flood-Nichols *et al.* [11] and Rodriguez *et al.* [12] found that Vitamin D deficiency during pregnancy was not associated with preterm birth.

Currently, there is not sufficient evidence showing whether vitamin D deficiency in pregnant women has beneficial or harmful effects on preterm birth. Hence, we carried out this meta-analysis to study the association between maternal vitamin D deficiency and PTB.

## 2. Methods

### 2.1. Data Sources

We performed a systematic literature search of Pubmed, Medline and the Cochrane Library through August 2015 for relevant articles using the following keywords: “vitamin D” or “cholecalciferol” or “25-hydroxyvitamin D” or “25(OH)D” in combination with “premature birth” or “preterm birth” or “PTB” or “preterm delivery” or “PTD” or “prematurity”. Additionally, we manually searched all eligible original articles, reviews and other relevant articles. This meta-analysis was performed following the guidelines for observation study protocols (MOOSE guidelines) [13].

### 2.2. Study Selection

Original articles exploring the relationship between maternal vitamin D status and pregnancy outcomes were reviewed and selected if they met the following inclusion criteria: (a) the study population was pregnant women without pre-chronic disease or HIV infection; (b) the study included women with singleton gestation; (c) participant age was ≥16 years; (d) maternal blood samples were taken for assays of 25(OH)D before or at delivery; (e) preterm birth was the outcome and the control group consisted of women without PTB; (f) the study presented sample sizes and odds ratios (OR) with 95% confidence intervals (CI) or information that could be used to infer these results; (g) vitamin D deficiency was defined as a 25(OH)D level below 20 ng/mL, and vitamin D insufficiency was defined as 25(OH)D levels of 21–29 ng/mL; (h) the study was published in English; and (i) the study met the predefined methodological quality assessment criteria for observational studies (supplementary materials Box S1). Studies with a score of 0 for any item or a total score <7 out of 10 maximal points were excluded [14].

Two reviewers (QLL and LBA) independently reviewed the electronic literature searches and acquired full-length articles for all citations meeting the predefined selection criteria. Final inclusion or exclusion decisions were made after reading the full text. We resolved any disagreements through consensus or arbitration by a third reviewer (XHL).

The following information was extracted from each study: the first author’s last name, year of publication, country of origin, study design, number of participants, diagnosis criteria of PTB, assay method of 25(OH)D, the prevalence of maternal vitamin D deficiency and the potential confounding factors included in the adjustments.

### 2.3. Statistical Analysis

We performed this-analysis with RevMan Software (Version 5.2, Cochrane Collaboration, London, UK). The association between maternal 25(OH)D levels and PTB was measured as an odds ratio (OR) and as the weight mean difference (WMD). If the OR and 95% CI were not available for the meta-analysis, these data were extracted from the selected articles to construct 2 × 2 tables of maternal low vitamin D status *versus* the presence or absence of adverse pregnancy outcomes. Results reported as the median and range were converted to the mean and standard deviation [15].

We used forest plots to visually assess pooled estimates and corresponding 95% CIs for each study. The heterogeneity among the results of the included studies was evaluated with *I*^2^ statistical tests. The results were considered statistically significant when *p* < 0.05. Once the effects were found to be heterogeneous (*I*^2^ > 50%), a random effects model was used. Otherwise, a fixed effect model was used.

Potential publication bias was examined using funnel plots. The sensitivity analysis was conducted not only to test the robustness of our results but also to investigate the effect of a single article on the overall risk estimated by removing one article in each turn. In addition, a subgroup analysis was performed to examine the possible reasons for heterogeneity. Two-tailed values of *p* < 0.05 were considered statistically significant.

## 3. Results

### 3.1. Search Results

**Figure 1:**
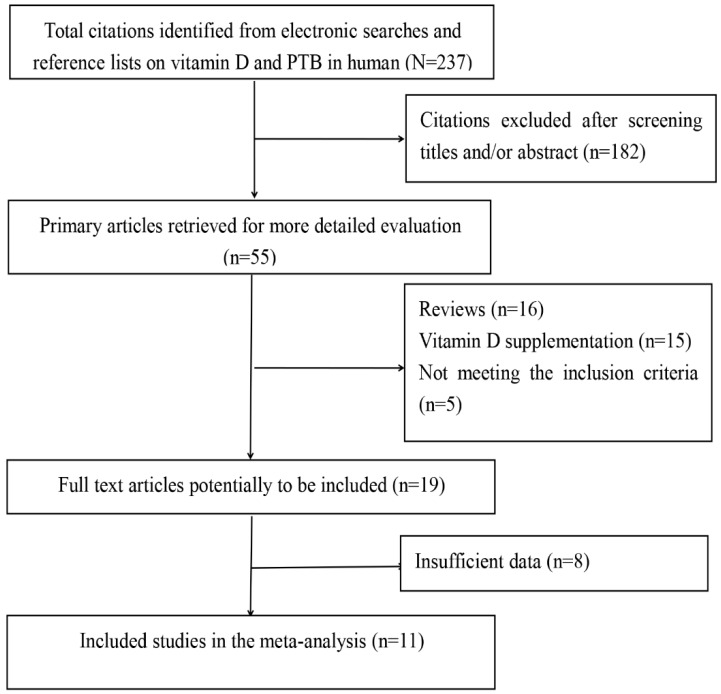
Flow chart of literature search and study selection.

Of the 237 records identified from our initial search, a total of 11 studies were finally identified through a strict screening process (Figure 1). The quality scores of these studies ranged from 9 to 10 according to the MOOSE guidelines, which indicates that all of the selected studies were of high quality (supplementary materials Table S1).

### 3.2. Characteristics of the Included Studies

The characteristics of the included articles in this meta-analysis are presented in Table 1. These studies were published from 2010 to 2015. Six studies were conducted in the US, two in Spain, and one each in Australia, China and Canada. One study had a cross-sectional design, one had a case-control design, one had a case-cohort design, one had a retrospective cohort design, four had nested case-control designs and four had prospective cohort designs. Moreover, five different assay techniques were used to measure maternal vitamin D levels and two different criteria were used for the diagnosis of PTB (<37 gestational weeks or <35 gestational weeks). Of these studies, 10 explored the association between maternal vitamin D deficiency and preterm birth, nine explored the association between maternal vitamin D insufficiency and preterm birth, and only six explored the mean difference in maternal vitamin D status among preterm birth and term birth. 

The diversity of participant characteristics was considerable in these studies. Out of a total of 10,098 participants, 2091 (20.7%) were diagnosed with PTB with consisted of different races. Additionally, the age of the pregnant women ranged from 16 to 40 years, and the mean BMI, if provided by studies, varied from 16.90 to 32.00 kg/m. In addition, the prevalence of vitamin D deficiency among the pregnant women varied from 6.9% to 59%.

### 3.3. Main Analysis

**Figure 2:**
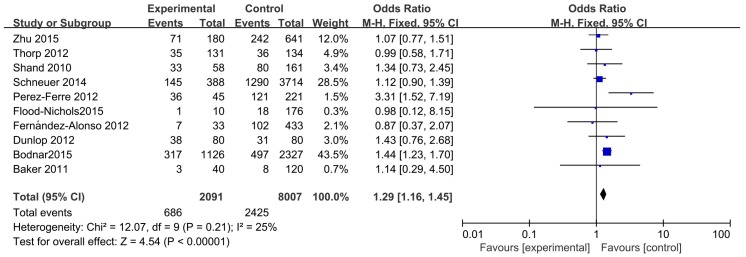
The meta-analysis of the association between maternal vitamin D deficiency and PTB.

The association between maternal vitamin D deficiency and the risk of preterm birth is presented in Figure 2 and supplementary materials Table S2. Ten studies involving 10,098 participants were included. As shown in this meta-analysis, the pregnant women with vitamin D deficiency (maternal serum 25 (OH) D levels < 20 ng/mL) had an increased risk of developing preterm birth (OR = 1.29, 95%CI: 1.16, 1.45) in the fixed-effects model. Based on nine studies, the pooled OR for vitamin D insufficiency (maternal serum 25(OH)D levels < 30 ng/mL) was calculated as 1.25 (95% CI: 1.11, 1.40) (supplementary materials Table S2 and Figure S1). Both results demonstrated that maternal vitamin D deficiency significantly increased the risk of PTB.

**Figure 3:**
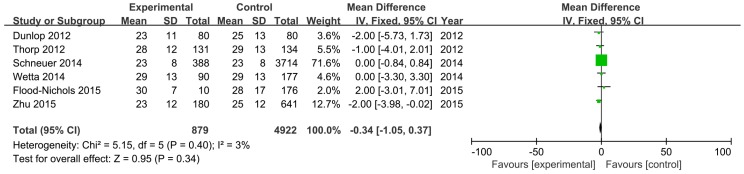
The meta-analysis of the association between maternal vitamin D level and PTB.

In Figure 3, we show a comparison of the mean difference between the PTB group (experimental group) and the control group in six studies involving 5801 participants. The pooled effect was −0.34 ng/mL (95% CI: −1.05, 0.37) with low heterogeneity (*I*^2^ = 3%, *p* = 0.39). Therefore, there was no statistically significant difference in maternal vitamin D levels between the PTB group and the control group.

### 3.4. Sensitivity and Subgroup Analysis

In order to explore the impact of various exclusion criteria on the overall risk estimate, sensitivity analysis and subgroup analysis were used to examine potential sources of heterogeneity in the meta-analysis. Bodnar’s study was responsible for most of the heterogeneity in this meta-analysis. Low heterogeneity was observed among the remaining studies (*I*^2^ = 8%, *p* = 0.37) and the pooled OR was 1.18 (95% CI: 1.01, 1.37) after excluding Bodnar’s study [8]. Furthermore, there were no obvious changes in the pooled ORs as a result of the exclusion of any other single study. The pooled ORs ranged from 1.18 (95% CI: 1.01, 1.37) to 1.36 (95% CI: 1.20–1.55), and each was statistically significant.

In the subgroup analysis with study design, we found that there was moderate heterogeneity in the prospective cohort studies (*I*^2^ = 60%, *p* = 0.06) (supplementary materials Figure S2). In the subgroup analysis with study country, the studies conducted in Spain were responsible for most of the heterogeneity (*I*^2^ = 80%, *p* = 0.02) (supplementary materials Figure S3). We did not conduct subgroup analyses of BMI, gestational week, race and season due to insufficient data in some studies.

### 3.5. Publication Bias

No obvious publication bias was observed in the funnel plots of this meta-analysis (supplementary materials Figure S4).

## 4. Discussion

The prevalence of vitamin D deficiency during pregnancy and its association with PTB have attracted much public health attention. In this meta-analysis, the results of 10 observational studies showed that maternal vitamin D deficiency was associated with an increased risk of PTB, while a comparison of the mean difference between the PTB group and the control group in six studies showed that there was no statistical difference by maternal vitamin D levels. The result of the pooled OR was more accurate and reliable than the pooled effect of WMD. Firstly, the diagnosis of vitamin D deficiency is more practically useful for clinical treatment than the exact vitamin D level. Secondly, in this meta-analysis, the conversion of the median and quartiles into the mean and standard deviation for the vitamin D levels, with a non-Gaussian distribution, reduced the precision of some studies [11,17]. Thirdly, five studies reported that maternal vitamin D levels were not associated with PTB, which may have an effect on the meta-analysis WMD results. Hence, we conclude that there was an association between maternal vitamin D deficiency during pregnancy and an increased risk of PTB. The result of our meta-analysis is consistent with previous studies. A meta-analysis of four observational studies showed that pregnant women with vitamin D deficiency experienced an increased risk of PTB (OR = 1.58, 95% CI: 1.08–2.31) [25]. Compared to this study, we provide stronger evidence of the association between maternal vitamin D deficiency and PTB, and we also conducted sensitivity and subgroup analyses to explore potential sources of heterogeneity.

From the funnel plots in this study, we conclude that there was no obvious publication bias. Furthermore, there was low heterogeneity in the meta-analysis for the pooled OR (*I*^2^ = 25%, *p* = 0.01). Our sensitivity analyses suggested that the study [8] conducted in Pittsburgh likely contributed to the heterogeneity. There may be other factors contributing to the heterogeneity in this meta-analysis. Firstly, there is still a lack of clear and uniform standards for defining vitamin D deficiency or insufficiency. Wetta *et al.* [18] defined vitamin D deficiency as a level of less than 15 ng/mL, while a level of less than 25 ng/mL was used in other studies. Secondly, there are several methods for measuring vitamin D, such as LC-MS (liquid chromatography-tandem mass spectrometry)and ELISA (electrochemiluminescence immunoassay). Thirdly, the time of blood sample collection varied from the first trimester to delivery. Fourthly, different seasons, the race of the pregnant women, diet during pregnancy and sunlight exposure during pregnancy are confounding factors for the association between maternal vitamin D and preterm birth. Consequently, more studies are needed to get more evidence to prove this finding.

In this meta-analysis, the prevalence of vitamin D deficiency was high among pregnant women although it may vary according to the latitude, ethnicity, supplementation of vitamin D, body mass index, season and the cut-off value used to define vitamin D deficiency. For pregnant women, on the one hand, vitamin D plays an important role in maintaining normal ranges of serum calcium and phosphorus by enhancing calcium absorption from the intestine and promoting the mobilization of calcium and other minerals from the skeleton [26]; on the other hand, vitamin D plays an immunomodulatory role in stimulating antimicrobial activity and enabling implantation [27]. Preterm birth is a heterogeneous syndrome, most commonly caused by infection and inflammation. Vitamin D, acting as an immune modulator, can reduce the possibility of preterm birth by inhibiting inflammation and regulating immune function.

Studies have reported that vitamin D could regulate both the acquired and innate immune responses at the fetal-maternal interface [28]. Vitamin D could function as an intracrine regulator of CAMP in trophoblasts to provide a novel mechanism for the activation of innate immune responses in the placenta [29,30]. 1,25-dihydroxyvitamin D is known to reduce bacterial infections by inducing cathelicidin in many tissues, including maternal and fetal cells of the placenta. A recent study found that vitamin D deficiency was strongly associated with bacterial vaginosis (BV) during pregnancy [31]. Adequate vitamin D status during pregnancy could reduce the risk of preterm delivery due to its decreasing placental colonization by bacterial vaginosis species [32,33]. Liu *et al.* [34] reported that the toll-like receptor triggers the vitamin D-mediated human antimicrobial response. Sufficient vitamin D can increase vitamin D receptor levels to produce antimicrobial peptides through the toll-like receptor pathway, while vitamin D deficiency could increase susceptibility to infection by impairing the induction of antimicrobial peptides in the same way. Vitamin D receptor polymorphism FokI was demonstrated to have an association with spontaneous idiopathic preterm birth in an Israeli population [35]. Therefore, maternal vitamin D during pregnancy has an effect on the prevention of preterm birth due to immune regulation and anti-inflammatory effects.

It is well known that the two major sources of vitamin D are exposure of the skin to solar ultraviolet B radiation and dietary intake. The cutaneous synthesis of vitamin D is greatly influenced by season, latitude, time of day, skin pigmentation, the amount of skin exposed, and whether makeup with sunscreen is used, so vitamin D levels vary among areas and persons. Vitamin D deficiency is typical in high-latitude areas and during winters characterized by short periods of sunlight, as seen in studies carried out in Pittsburgh, northern USA (40°N) [36] and the Hague, Netherlands (52°N) [37]. Therefore, dietary vitamin D supplementation is a feasible way for pregnant women to maintain sufficient vitamin D levels.

Few trials of vitamin D supplementation have been conducted in pregnant women with adequate power to test its effects on birth outcomes. However, the preventive effect of vitamin D supplementation on preterm labor is controversial. Some studies report that maternal supplementation with vitamin D during pregnancy is associated with a reduced risk of preterm birth. For instance, Wagner *et al.* showed that achieving a 25(OH)D serum concentration ≥40 ng/mL could significantly decrease the risk of PTB compared to concentrations ≤20 ng/mL after maternal supplementation with vitamin D during pregnancy [38,39], while other studies have obtained different results. Thus, large, randomized controlled trials focusing on reducing PTB and its consequences are needed to accurately evaluate the potential benefits of these low-cost interventions in the future.

There were several limitations in this meta-analysis. Firstly, different diagnostic criteria, resulting in different threshold values, for preterm birth and vitamin D deficiency, insufficiency or sufficiency could have influenced the pooled effect. Two definitions of vitamin D deficiency (<20 ng/mL or <15 ng/mL) and two diagnostic criteria for preterm birth (≤37 gestational weeks or ≤35 gestational weeks) were used in this meta-analysis. The data were pooled based on the cut-off value of less than 20 ng/mL for vitamin D deficiency, and preterm birth was classified per the definition used in the included studies. Thus, different diagnostic criteria of preterm birth and vitamin D deficiency, insufficiency or sufficiency could have influenced the pooled effect. Secondly, different assay techniques were used to measure maternal vitamin D levels. Thirdly, the observed association between maternal vitamin D and preterm birth could be affected by confounding factors, such as race, sunlight exposure and BMI. Some, but not all, of the individual studies generated adjusted OR, so we could not pool the findings by adjusting for confounding factors. Fourthly, only published articles were included.

## 5. Conclusions

The evidence presented in this article indicates an association between vitamin D deficiency and an increasing risk of preterm birth. Pregnant women with serum vitamin D levels less than 20 ng/mL experience an increasing risk of preterm birth (OR = 1.29; 95% CI: 1.16, 1.45). However, more studies are needed to better understand the effect of maternal vitamin D on preterm birth, and well-designed trials are required to determine the explicit effect of vitamin D supplementation on the prevention of preterm birth. The prevention of preterm birth is a global priority; therefore, screening women who are at risk of vitamin D deficiency and determining how to supplement with vitamin D could be considered in the future.

## Figures and Tables

**Table 1:** Characteristics of observational studies included in this meta-analysis.

Author and Year	Country	Study Design	Sample Size (n)	PTB (*n*)	PTB Criteria (GW)	Assay Method	Mean 25(OH)Dng/mL(SD) PTB NPTB		Prevalence	Significant	Adjustment
Bodnar (2015) [8]	US	Case–cohort	12,861	1126	<37	LC-MS	NA	NA	11.3%	Yes	a,b,c,d,e,f,g,h,i,j,l
Flood-Nichols (2015) [11]	US	Retrospective	235	10	<37	ELISA	30 ± 7	27 ± 17	10%	No	a,b,c,d
Zhu (2015) [16]	China	Prospective	821	180	<37	ELISA	23 ± 12	25 ± 13	NA	Yes	No
Schneuer (2014) [17]	Australian	Nested case-control	5109	388	<37	AIA	23 ± 8	23 ± 8	NA	No	b,d,f,g,l,m,n
Wetta (2014) [18]	US	Nested case-control	177	90	<35	LC-MS	29 ± 13	29 ± 13	NA	No	b,e,f,l,n,o
Fernández-Alonso (2012) [19]	Spain	Prospective	466	33	<37	ECLIA	NA	NA	23.4%	No	No
Perez-Ferre (2012) [20]	Spain	Prospective	266	45	<37	CLIA	NA	NA	59%	Yes	c,n,o
Dunlop (2012) [21]	US	Cross-sectional	160	80	<37	ELISA	23 ± 11	25 ± 13	NA	No	a,e,g,n,q
Thorp (2012) [22]	US	Nested case-control	134	131	<35	LC-MS	28 ± 12	29 ± 13	22%	No	e,p
Baker (2011) [23]	US	Nested Case-Control	160	40	<37	LC-MS	NA	NA	6.9%	No	a,b,g,h,n
Shand (2010) [24]	Canada	Prospective	219	58	<37	RIA	NA	NA	53%	No	a,b,c,d,f,n,r

PTB: preterm birth; NPTB: non preterm birth; GW: gestational week; AIA: automated immunoassay; CLIA: chemiluminescence immunoassay; ELISA: electrochemiluminescence immunoassay; LC-MS: liquid chromatography-tandem mass spectrometry; RIA: radioimmunoassay; NA: not available; Prevalence: prevalence of maternal vitamin D deficiency; a: BMI; b: season; c: ethnicity; d: smoking; e: race; f: parity; g: socioeconomic disadvantage; h: gestational age of blood sampling; i: assay batch; j: year of delivery; l: weight; m: control of birth; *n*: age; o: previous gestational history; p: study center; q: marital status; r: multivitamin use.

## Supplementary Materials

Supplementary materials can be found at http://www.mdpi.com/2072-6643/8/5/301/s1.
