# Supplementary material for: Does Maternal Vitamin D Deficiency Increase the Risk of Preterm Birth: A Meta-Analysis of Observational Studies
Source: Nutrients. 2016 May 20;8(5):301. doi: 10.3390/nu8050301 (PMC4882713; doi:10.3390/nu8050301)
Supplement: Supplementary file 1 [file nutrients-08-00301-s001.docx]

Supplementary Materials: Does Maternal Vitamin D Deficiency Increase the Risk of Preterm Birth: A Meta-Analysis of Observational Studies

Lu-Lu Qin, Hui-Lan Xu and Bang-An Luo

**Box S1.** Quality assessment of observational studies (total 10 points) *.

| 1. Selection of Participants (1/0)  Cohort studies (1/0)  Selected cohort was representative of the general population (population-based studies) or target catchment population (hospital-based studies) (1)  Cohort was a selected unrepresentative group (0)  Case control studies (1/0)  Cases and controls drawn from the same population (1)  Cases and controls drawn from different sources or the selection of groups (0)  2. Comparability of Groups (2/0)  No significant differences between the groups reported in terms of age, plurality, smoking, history of preterm birth, preeclampsia or gestational diabetes, pre-existing medical conditions were explicitly reported, or these differences were adjusted for (2)  Differences between groups were not examined (1)  Groups differed and no adjustment results provided (0)  3. Definition of Outcomes (2/0)  Definition of outcomes  Referenced or standard definition (2)  Explicit non-standard definition (1)  Unspecified or unacceptable definition (0)  4. Ascertainment of Outcomes (2/0)  How the diagnosis was made  Prospectively diagnosed or review of notes/hospital discharge records (2)  Retrospective chart review or database coding (1)  Process not described (0)  5. Sample Size (1/0)  ≥200 participants in a cohort study; ≥50 participants in either group (case/control) (1)  100≤ participants <200 in a cohort; 25≤ participants <50 in either group (case/control) (0.5)  Participants <100 or total number of events <10 in a cohort; participants <25 in either group (case/control) (0)  6. Study Design (2/0)  Prospective cohort or nested case-control within a prospective cohort (2)  Cross-sectional, case-control or retrospective cohort (1)  Not described or poorly designed (0)  **Exclusion:** score zero in any item (1 to 6) or a total score <7 out of 10 maximal points |
| --- |

* A score based quality assessment criteria for non-randomized observational studies adapted from Duckitt & Harrington.

**Table S1.** Quality scores of included studies on vitamin D status and pregnancy outcomes.

| **Study** | **Selection of Participants** | **Comparability of Groups** | **Outcomes Definition** | **Ascertainment** | **Sample Size** | **Study Design** | **Total Score** |
| --- | --- | --- | --- | --- | --- | --- | --- |
| Bodnar (2015) | 1 | 2 | 2 | 2 | 1 | 1 | 9 |
| Flood-Nichols (2015) | 1 | 2 | 2 | 2 | 1 | 1 | 9 |
| Zhu (2015) | 1 | 1 | 2 | 2 | 1 | 2 | 9 |
| Schneuer (2014) | 1 | 2 | 2 | 2 | 1 | 2 | 10 |
| Wetta (2014) | 1 | 2 | 2 | 2 | 1 | 2 | 10 |
| Fernández-Alonso (2012) | 1 | 1 | 2 | 2 | 1 | 2 | 9 |
| [Perez-Ferre](http://www.ncbi.nlm.nih.gov/pubmed/?term=Perez-Ferre%20N%5bAuthor%5d&cauthor=true&cauthor_uid=22548949) (2012) | 1 | 2 | 1 | 2 | 1 | 2 | 9 |
| Dunlop (2012) | 1 | 2 | 2 | 2 | 1 | 1 | 9 |
| Thorp(2012) | 1 | 2 | 2 | 2 | 1 | 2 | 10 |
| Baker (2011) | 1 | 2 | 1 | 2 | 0.5 | 2 | 9.5 |
| Shand(2010) | 1 | 2 | 2 | 2 | 1 | 2 | 10 |

**Table S2.** Results of meta-analysis according to 25(OH)D level.

| **Vitamin D Status** | **25(OH)D ng/mL** | **PTB(*n*)** | **NPTB(n)** | **OR** | **95% CI** | ***I*^2^(%)** | ***p*** |
| --- | --- | --- | --- | --- | --- | --- | --- |
| Deficiency | <20 | 2091 | 8007 | 1.29 | 1.16, 1.45 | 25 | 0.21 |
| Insufficiency | <30 | 1970 | 7970 | 1.25 | 1.11, 140 | 0 | 0.81 |


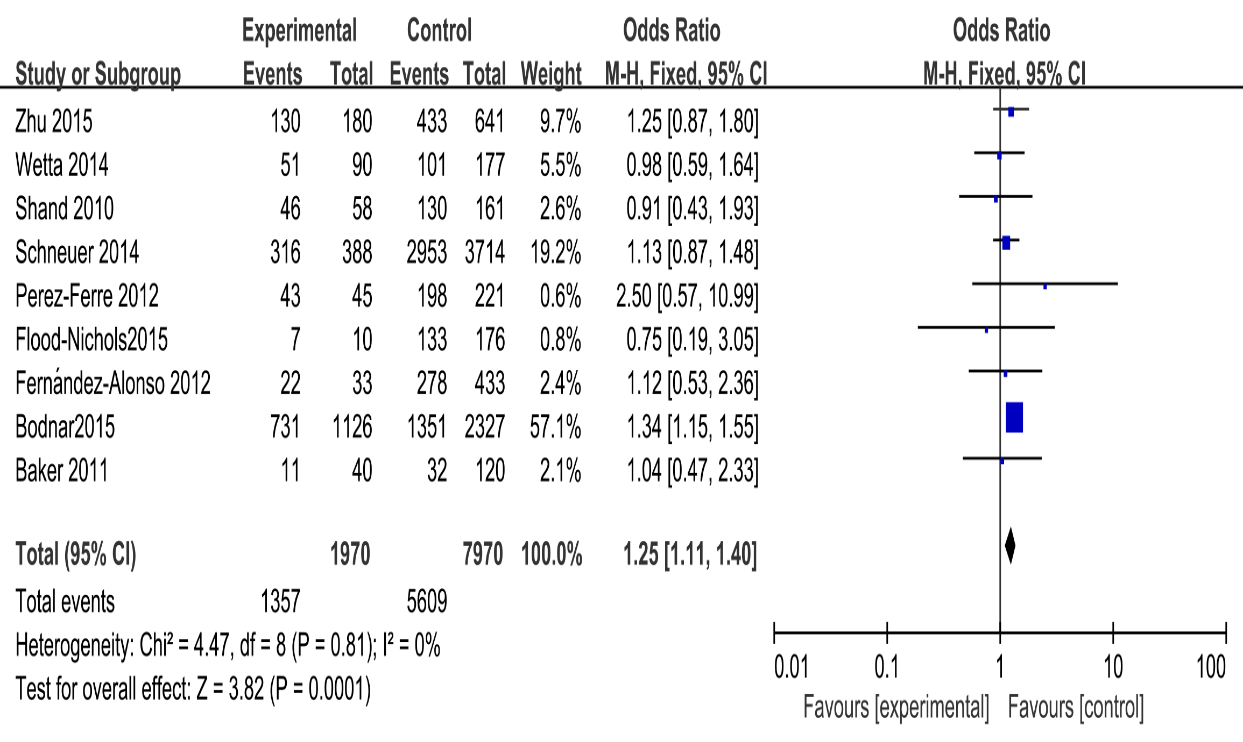


**Figures S1.** The meta-analysis of the association between maternal vitamin D insufficiency (<30 ng/mL) and PTB.


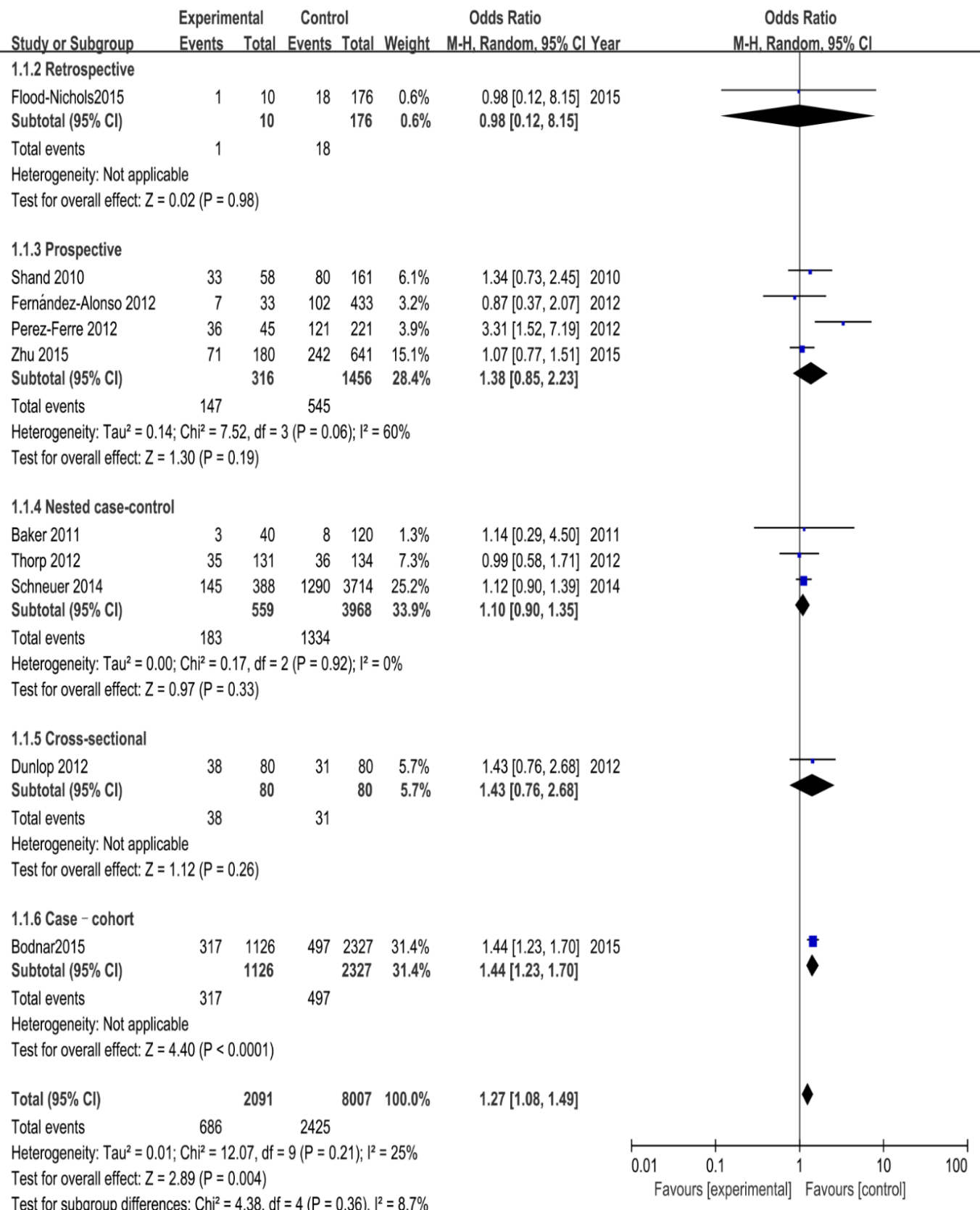


**Figure S2.** The results of subgroup analysis according to study design.


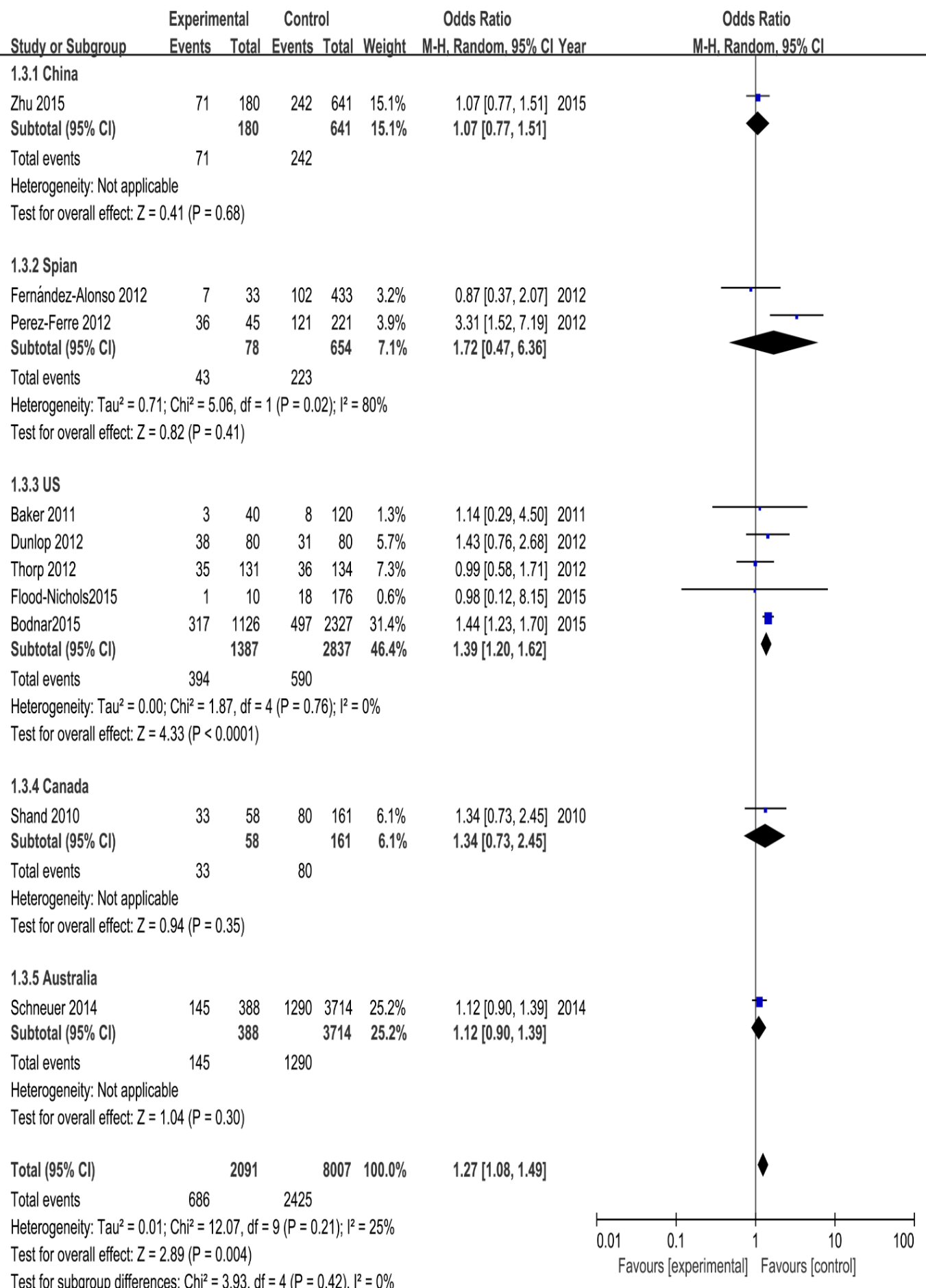


**Figure S3.** The results of subgroup analysis according to different country.

**
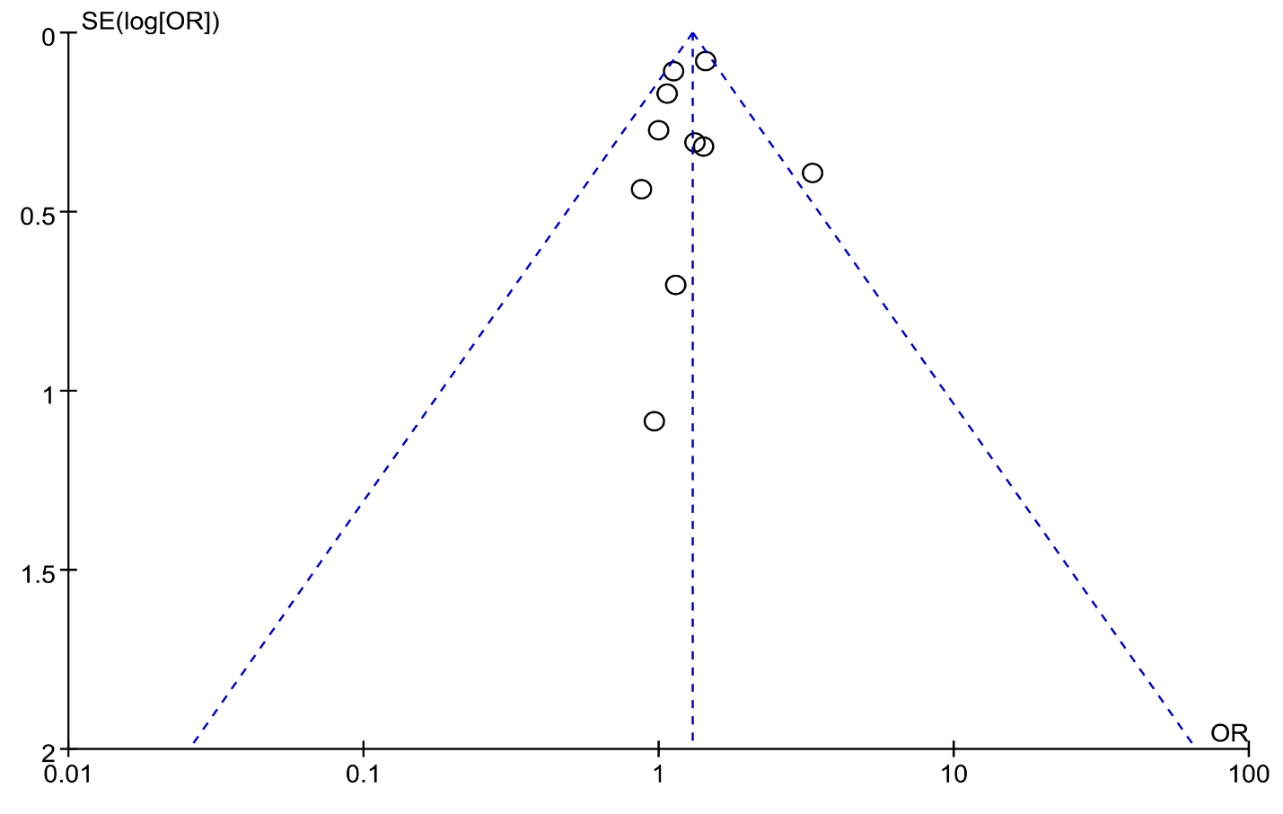
**

**Figure S4.** The result of Funnel plots.
